# WRKY61 Negatively Regulates Aluminum Resistance by Inhibiting the Expression of *ALMT1* in *Arabidopsis thaliana*

**DOI:** 10.3390/plants14213286

**Published:** 2025-10-27

**Authors:** Aolin Ma, Jie Li, Siqi Liu, Zhixuan Du, Jianjun Zeng, Yonghong Xiao, Guanping Feng

**Affiliations:** 1 Key Laboratory of Jiangxi Province for Biological Invasion and Biosecurity, School of Life Sciences, Jinggangshan University, Ji’an 343009, China; 2 Key Laboratory of Jiangxi Province for Functional Biology and Pollution Control in Red Soil Regions, School of Life Sciences, Jinggangshan University, Ji’an 343009, China

**Keywords:** aluminum resistance, *ALMT1*, WRKY61, transcriptional repressor

## Abstract

Aluminum (Al) toxicity is a major constraint on crop production in acidic soils. A key mechanism for aluminum resistance in many plants involves the ALMT1-mediated exudation of malate from the root system. This process hinges on the precise regulation of *ALMT1* expression, which is therefore critical for plant tolerance to aluminum toxicity. In a screen for Arabidopsis mutants with altered aluminum resistance, we found that the loss-of-function mutant of the WRKY61 transcription factor exhibited significantly enhanced resistance to aluminum toxicity, indicating that WRKY61 is involved in the plant’s response to aluminum toxicity. Further research revealed that WRKY61 binds to the W-box in the *ALMT1* promoter to repress its expression. Mutation of WRKY61 resulted in increased malate secretion from mutant roots, which chelated aluminum ions, leading to a significant reduction in aluminum content within the plant. This, in turn, significantly enhances malate secretion under aluminum toxicity, ultimately conferring heightened aluminum resistance. These results clearly indicate that WRKY61, as a transcriptional repressor of *ALMT1*, plays a negative regulatory role in plant resistance to aluminum toxicity.

## 1. Introduction

Globally, agricultural productivity faces a major constraint in the form of soil acidification (pH < 5.5). This condition triggers the release of free trivalent aluminum (Al^3+^) into the soil solution. In acidic soils, Al^3+^ severely inhibits root tip cell elongation, impairing water and mineral nutrient uptake. Consequently, aluminum stress constitutes a primary constraint on plant growth in these soils, which cover 30–40% of the planet’s potential farmland [1]. To counteract the harmful effects of aluminum (Al), plants have developed two main resistance strategies: exclusion and accumulation [2,3,4]. The exudation of organic acids, notably malate, citrate, and oxalate, represents the most thoroughly characterized and essential strategy for Al exclusion by plants [2,3,5]. The accumulation mechanisms, originally characterized in species capable of hyper-accumulating Al (like hydrangea, buckwheat, and tea), rely on the intracellular compartmentalization or internal sequestration of aluminum. Recent findings reveal that Arabidopsis thaliana and rice detoxify aluminum internally via vacuolar sequestration, a process involving Al absorption and its compartmentalization within vacuoles [6,7,8,9].

In response to aluminum (Al) toxicity, root tips exude organic acid anions into the rhizosphere, where these anions form complexes with Al^3+^, thereby inhibiting their uptake and mitigating damage to the root system. The root apex, being the most Al-sensitive region, is particularly vulnerable to Al toxicity. Maize and soybean primarily rely on citrate, although they might also release malate as a defense mechanism. In contrast, wheat and oilseed rape exude both malate and citrate to combat Al toxicity. In Arabidopsis, root-mediated Al resistance heavily relies on the ALMT1 transporter facilitating malate exudation, and the MATE transporter facilitating citrate exudation. Notably, ALMT1-dependent malate release contributes more quantitatively than MATE-dependent citrate release [10,11].

Studies indicate that the regulation of *ALMT1* in response to aluminum involves intricate signaling networks, including those mediated by phytohormones (e.g., auxin, cytokinin, jasmonate, ABA) and ROS [12,13]. *ALMT1* transcription was clearly induced by indole-3-acetic acid (IAA), abscisic acid (ABA), low pH, and hydrogen peroxide [12,13]. The C_2_H_2_-type zinc finger transcription factor STOP1 is crucial for modulating Al resistance through the direct regulation of downstream Al-resistance gene expression, notably ALMT1 and MATE [14]. Several studies highlight the diverse post-transcriptional and post-translational pathways contributing to the regulation of STOP1. These processes include nuclear mRNA export and protein modifications such as ubiquitination, SUMOylation, phosphorylation, and oxidation [15,16,17]. According to recent findings, calcium-protein kinases (CPKs) help plants effectively respond to and adapt to aluminum toxicity. Al-activated CPK21, CPK23, and CPK28 phosphorylate STOP1, enhancing its nuclear entry and preventing its degradation through inhibition of its interaction with the F-box protein RAE1 [18,19]. Furthermore, STOP1 and CAMTA2 are involved in the Al-inducible expression of ALMT1, and both proteins bind to the *ALMT1* promoter. The regulation of *ALMT1* expression involves CML24 physically interacting with CAMTA2. Their synergistic action then counteracts the repression of *ALMT1* by interacting with the transcriptional suppressor WRKY46, which functions as a transcriptional repressor of *ALMT1* [20,21]. Given ALMT1’s responsiveness to diverse hormones and environmental signals, its expression regulation is highly complex, warranting further investigation.

The transcription factor WRKY family is a major group of plant transcription factors, with 72 known members in Arabidopsis. Their proteins share a conserved WRKY domain on the N-terminus and an atypical zinc finger structure on the C-terminus [22,23]. Recent studies consistently emphasize the crucial role of WRKY transcription factors in managing plant stress responses and influencing plant-specific growth and development [24]. In a screen for Arabidopsis mutants exhibiting abnormal responses to aluminum toxicity, we identified that the loss-of-function mutant of WRKY61 displayed significantly enhanced resistance compared to the wild-type control. This observation suggests that WRKY61 is involved in the plant’s response to aluminum toxicity. Further investigation revealed that WRKY61 inhibits the expression of *ALMT1*, which itself functions as a negative regulator of aluminum resistance.

## 2. Results

### 2.1. Wrky61-1 Mutant Shows Increased Resistance to Aluminum Toxicity

**Figure 1:**
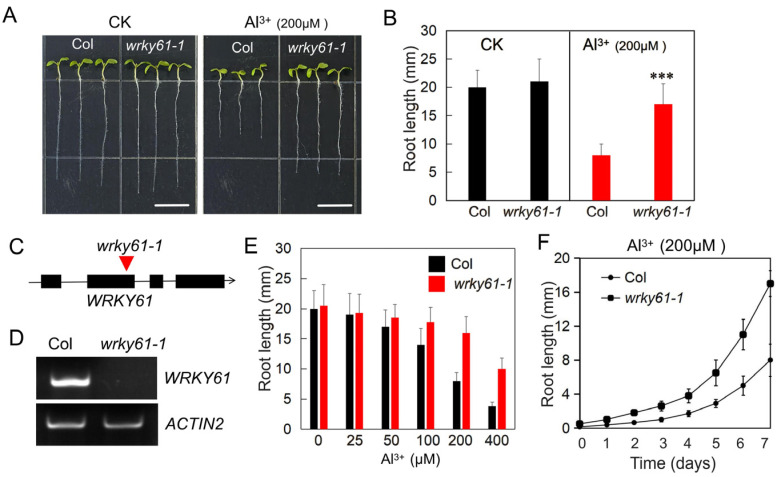
*wrky61-1* mutant exhibited enhanced aluminum resistance. (**A**) Phenotypes of wild-type (Col) and *wrky61-1* mutant plants grown on solid medium, with or without 200 µM AlCl_3_ treatment (pH 5.0). Scale bar = 5 mm. CK, the blank control. (**B**) The length of root of the seedling in (**A**). Data are presented as the mean ± SE (n > 20). Asterisks indicate statistically significant differences (***, *p* < 0.01). (**C**) Schematic diagram of *WRKY61* and *wrky61-1* mutants. (**D**) Semi-quantitative PCR was performed to detect the expression of *WRKY61* in the *wrky61-1* mutant, with *ACTIN2* as the internal reference gene. (**E**) Effects of different Al^3+^ concentration treatments on root length in wild-type (Col) and *wrky61-1* mutant. Data are presented as the mean ± SE (n > 20). (**F**) Root length curves of Col and *wrky61-1* mutant plants under 200 μM AlCl_3_ treatment for different days. Data are presented as the mean ± SE (n > 20).

To identify key genes involved in plant resistance to aluminum toxicity, we screened Arabidopsis mutants in a 1/2 MS medium containing 200 μM AlCl_3_ at pH 5.0, a concentration that significantly inhibits seedling growth, as confirmed by the dose–response analysis (Figure 1). This process yielded several mutants with significantly stronger resistance to aluminum toxicity than the wild-type control, one of which was the *wrky61-1* mutant, a loss-of-function mutant of the WRKY61 transcription factor. Aluminum toxicity severely inhibited the root growth of the wild-type (WT) Col-0, resulting in a 65% reduction in root length. In contrast, the *wrky61-1* mutant exhibited significantly greater resistance, with only a 19% reduction in root length (Figure 1A,B). Another loss-of-function mutant of WRKY61, *wrky61-2*, also exhibited significant resistance to aluminum stress (Figure S1). The *wrky61-1* mutant (SALK_006029C) and *wrky61-2* mutant (GABI_561F07) were obtained from the NASC mutant stock center, and the T-DNA insertions are located in the second exon and the third exon, respectively. Semi-quantitative RT-PCR analysis indicated that both of the mutants are the knockout mutants (Figure 1C,D and Figure S1). Consistent with these findings, root growth curves revealed that while the mutant’s germination was unaffected by the 200 μM Al^3+^ treatment, its subsequent growth rate was markedly lower than that of the WT (Figure 1F). Collectively, these results demonstrate that the *wrky61-1* mutant possesses a high level of resistance to aluminum toxicity.

### 2.2. Reduced ROS Accumulation Under Aluminum Toxicity in Wrky61-1 Mutant

**Figure 2:**
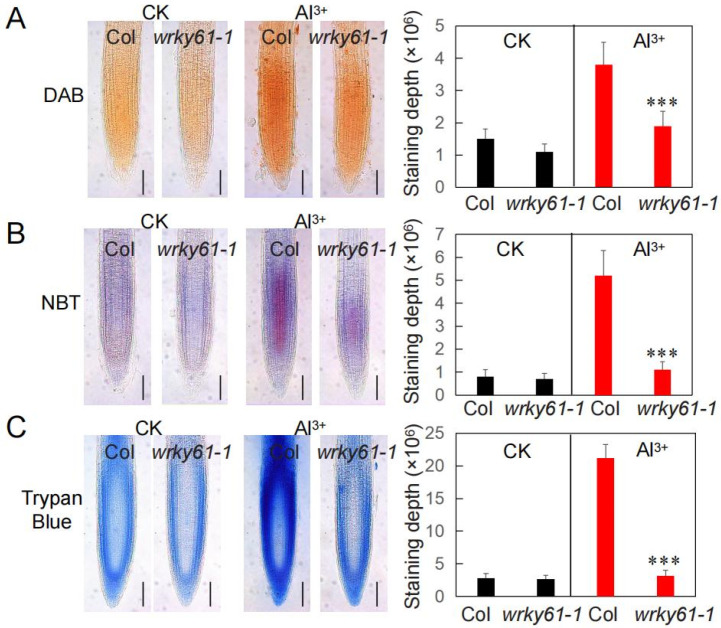
Observation of ROS accumulation and cell death in the roots under aluminum toxicity. The primary root images and staining intensity of DAB staining (**A**), NBT staining (**B**), and trypan blue staining (**C**) for 1 h with 200 µM AlCl_3_ treatment (pH 5.0). CK, the blank control. Scale bar = 100 µm. Data are presented as the mean ± SE (n > 20). Asterisks indicate statistically significant differences (***, *p* < 0.01).

When plants are under stress, they produce reactive oxygen species (ROS), which forces a shift from growth to defense. 3,3′-diaminobenzidine (DAB) staining revealed that H_2_O_2_ overaccumulation in root tips after 1 h of 200 μM Al^3+^ treatment was significantly attenuated in the *wrky61-1* mutant compared to the control. While a 1 h treatment with 200 μM Al^3+^ induced superoxide (O_2_^−^) accumulation in wild-type Arabidopsis roots, nitro blue tetrazolium (NBT) staining revealed that the O_2_^−^ level was significantly decreased in the *wrky61-1* mutant (Figure 2A,B). To assess the impact of Al toxicity on cell viability, Arabidopsis roots were stained with trypan blue. The results indicated that a 1 h Al^3+^ treatment induced significant cell death in WT roots, a response that was markedly attenuated in the *wrky61-1* (Figure 2C). These findings demonstrate that mutation of *WRKY61* confers increased tolerance to aluminum toxicity.

### 2.3. WRKY61 Negatively Regulates the Expression of ALMT1

**Figure 3:**
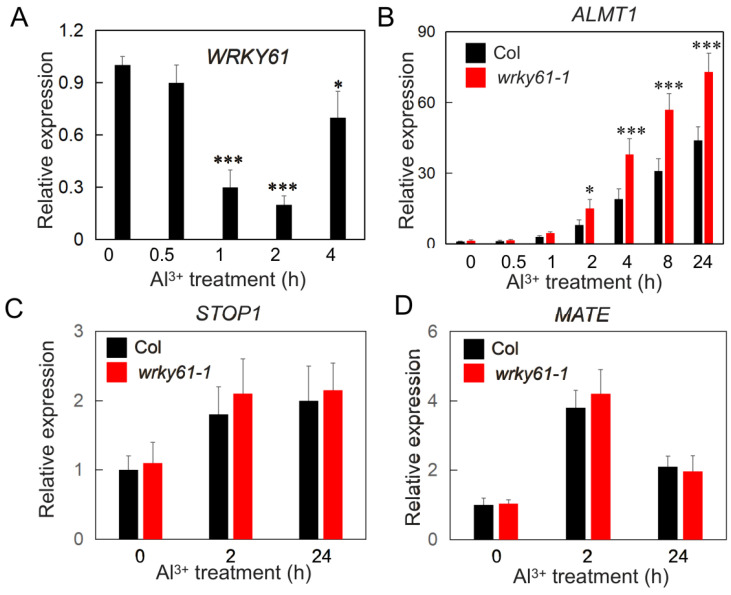
WRKY61 suppresses *ALMT1* expression. (**A**) WRKY61 expression was analyzed in 7-day-old Col-0 roots after treatment with 200 µM AlCl_3_. RNA was extracted from control and Al-treated samples. The data show one of three independent experiments and error bars represent the standard error. Asterisks indicate statistically significant differences (*, *p* < 0.05; ***, *p* < 0.01). (**B**) *ALMT1* expression in Col and the *wrky61–1* mutant following 200 µM AlCl_3_ treatment (pH 5.0) over various time points. The data show one of three independent experiments and error bars represent the standard error. Asterisks indicate statistically significant differences (*, *p* < 0.05; ***, *p* < 0.01). (**C**,**D**) *STOP1* or *MATE* expression in Col and the *wrky61-1* mutant following 200 µM AlCl_3_ treatment (pH 5.0) over various time points. The data show one of three independent experiments and error bars represent the standard error.

The results of quantitative real-time PCR (qRT-PCR) showed that the expression of *WRKY61* was significantly suppressed after 1 h of treatment with 200 μM Al^3+^. However, this inhibitory effect became less pronounced after 4 h of treatment (Figure 3A). The results demonstrate that aluminum stress modulates WRKY61 expression in plants, which may facilitate active adaptation to aluminum stress.

Under aluminum toxicity, plants secrete organic acids from their roots to chelate aluminum ions, thereby mitigating the adverse effects on their growth and development. In Arabidopsis, resistance to Al toxicity is achieved through the ALMT1-dependent exudation of malate from the roots. We hypothesized that the WRKY61 transcription factor acts on the malate secretion, thereby affecting plant resistance to aluminum toxicity. To test this, we used qRT-PCR to examine the expression of *ALMT1* in the *wrky61-1* mutant background. We found that under aluminum toxicity, the expression level of *ALMT1* in the mutant was significantly higher than in the wild-type control (Figure 3B). This result suggests that WRKY61 regulates the expression of *ALMT1* and is involved in the plant’s response to aluminum toxicity. As a C_2_H_2_-type zinc finger transcription factor, STOP1 confers aluminum resistance by directly binding to and regulating downstream genes such as ALMT1 and MATE, which encodes a root citrate transporter. We further examined the expression of *STOP1* and *MATE* (AT1G51340) in the mutant under aluminum toxicity and found that their expression levels were almost identical to those in the wild-type (Figure 3C,D). This suggests that the function of WRKY61 may be specific to regulating *ALMT1* expression. These results show that WRKY61 affects the expression of the malate transporter ALMT1.

### 2.4. WRKY61 Binds to the W-Box in the ALMT1 Promoter

**Figure 4:**
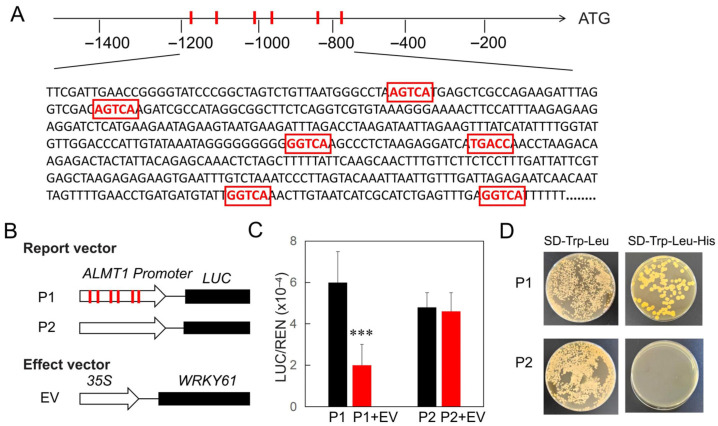
WRKY61 binds to the W-box in the *ALMT1* promoter. (**A**) The *ALMT1* promoter was characterized, and potential W-boxes were identified based on the presence of the TGACC/T motif. (**B**) For the transient expression assay in *N. benthamiana*, the full-length and W-box–deleted *ALMT1* promoters were cloned into the reporter vector, and the WRKY61 coding sequence was cloned into the effector vector. (**C**) LUC/REN Ratios. Firefly luciferase (LUC) activity was normalized to Renilla luciferase (REN) activity, which served as an internal control. Data are presented as the mean ± standard error (SE) from three biological replicates. Asterisks indicate statistically significant differences (***, *p* < 0.01). (**D**) WRKY61 binds to the *ALMT1* promoter in yeast. Yeast cells were co-transformed with a bait vector (containing a promoter fragment P1 or P2 fused to the HIS2 reporter gene) and a prey vector (expressing WRKY61-GAL4 activation domain). Transformants were grown in liquid medium and spotted onto dual-selection plates (SD/-Trp/-Leu) and interaction-selection plates (SD/-Trp/-Leu/-His).

WRKY transcription factors, characterized by one or two conserved WRKY domains (WRKYGQK) at their N-termini and zinc-finger-like motifs at their C-termini, exhibit specific affinities for binding to W-boxes (TTGAC[T/C]) within the promoter regions of their target genes. Whether WRKY61 directly binds to the ALMT1 promoter to repress its expression remains to be determined. To this end, we first analyzed the *ALMT1* promoter and identified six W-boxes within the upstream 500 bp to 1200 bp region of ATG, indicating that *ALMT1* is a potential target gene of WRKY transcription factors (Figure 4A). To test this, we first used agro-infiltration to transiently transform Nicotiana benthamiana leaves, a well-established method for analyzing plant promoters and transcription factors. Using a dual-luciferase assay, we co-expressed the intact (P1) and W-box-deleted (P2) *ALMT1* promoter-reporter constructs with a 35S: WRKY61 effector vector (EV) in Nicotiana benthamiana (Figure 4B). Notably, reporter activity driven by the full-length *ALMT1* promoter was inhibited by WRKY61, whereas no such inhibition was observed with the W-box-deleted promoter (Figure 4C). Additionally, we employed the yeast one-hybrid assay to determine whether WRKY61 binds to the W-BOX region of the *ALMT1* promoter. Yeast co-transformed with WRKY61 and the intact P1 *ALMT1* promoter-reporter construct grew well in selective medium, whereas co-transformation with WRKY61 and the W-box-deleted P2 construct did not (Figure 4D). This indicates WRKY61 binds the *ALMT1* promoter specifically through W-boxes. The results demonstrate that the W-boxes are necessary for the functional interaction between WRKY61 and the *ALMT1* promoter.

### 2.5. WRKY61 Suppresses Malate Secretion

**Figure 5:**
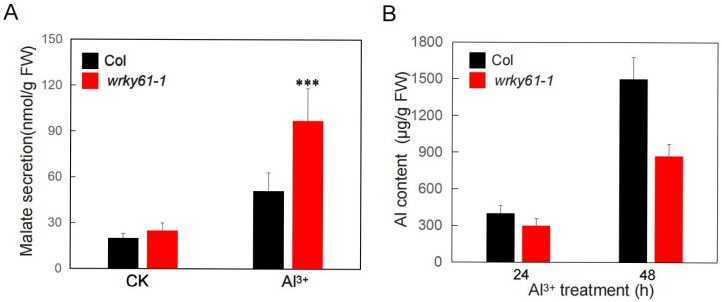
(**A**) Malate secretion from Col and *wrky61-1* roots. 2-week-old seedlings were exposed to a 0.5 mM CaCl_2_ solution (pH 5.0) with or without 200 µM AlCl_3_. FW, fresh weight. Data are from three independent biological replicates. Error bars indicate the standard error of the mean. Asterisks indicate statistically significant differences (***, *p* < 0.01). (**B**) Aluminum (Al) accumulation analysis in Col and *wrky61-1* seedlings. Data are from three independent biological replicates. Error bars indicate the standard error of the mean.

Given that ALMT1 expression is altered in the mutant, we further compared malate secretion between wild-type and *wrky61–1* plants under both normal and Al treatment conditions. After hydroponic culture for 4 weeks, plants of both genotypes were treated with or without 200 µM AlCl_3_ for 12 h. HPLC-MS/MS analysis revealed no significant difference in malate secretion between the *wrky61-1* mutant and the wild-type under non-aluminum toxicity. However, under aluminum toxicity, the mutant secreted over 80% more malate than the wild-type (Figure 5A). Additionally, we employed ICP-AES to measure the aluminum content in Arabidopsis seedlings grown in 1/2 MS medium for 10 days treated with or without 200 µM AlCl_3_ (Figure 5B). The results revealed that the aluminum content in the *wrky61–1* mutant was significantly lower than that in the wild-type controls, reaching only 57% of the wild-type. The results indicated that WRKY61 suppresses the secretion of malate in the roots under aluminum toxicity.

## 3. Discussion

Across diverse plant species, WRKY transcription factors are involved in a great many vital processes, notably seed development and germination, senescence, and the management of biotic and abiotic stresses [24,25]. An accumulating body of evidence indicates that WRKY transcription factors, functioning as substrates for diverse kinases and E3 ubiquitin ligases, are integral to the regulation of plant stress resistance, growth, and development. These findings imply a significant role for WRKY transcription factors in modulating the balance between plant growth and defense responses [26,27,28]. In *Arabidopsis thaliana*, WRKY7, WRKY11, WRKY15, WRKY17, WRKY21, and WRKY39 are recognized for their dual roles in growth and drought tolerance, which are activated in response to drought stress [24]. WRKY61 belongs to the WRKY IId subfamily. Previously, Wang’s research showed that WRKY61 exhibited significant upregulation in Arabidopsis infected with Turnip crinkle virus (TCV), and higher expression of WRKY61 reduces TCV viral accumulation [29]. Screening Arabidopsis mutants for altered aluminum resistance led to the identification of the *wrky61-1* mutant, which showed enhanced aluminum resistance. WRKY61 expression was found to decrease under aluminum toxicity. We determined that WRKY61 binds to the W-BOX of the *ALMT1*’s promoter, inhibiting its transcription. The mutation of WRKY61 removes this inhibition, enabling increased *ALMT1* expression and subsequent malate secretion under aluminum toxicity, which enhances plants’ aluminum resistance. While WRKY61 enhances viral resistance in plants, it paradoxically compromises aluminum tolerance.

Al-resistant plants utilize the secretion of organic acids from their roots as a vital strategy to detoxify external aluminum. The malate transporter ALMT1, known for conferring Al resistance, was first identified in wheat; homologous versions have also been isolated in Arabidopsis and oilseed rape [30,31,32]. While STOP1 directly regulates ALMT1 expression by binding to its promoter, the transcription factor itself remains unresponsive to aluminum toxicity. This suggests that other Al-responsive factors contribute to the regulation of ALMT1 expression [19]. Unlike STOP1, *ALMT1* expression is significantly upregulated by aluminum toxicity and is also strongly induced by various signals, including IAA, ABA, low pH, hydrogen peroxide, and flg22 [12,13]. In this work, we show that WRKY61 serves as a transcriptional repressor of *ALMT1*, playing a role in the modulation of Arabidopsis Al tolerance. Nevertheless, it is still unclear how WRKY61 integrates plant hormone and environmental signals to modulate *ALMT1* expression levels, consequently adjusting the equilibrium between plant growth and defense responses.

W-boxes (TTGAC[T/C]), recognized as binding sites for WRKY proteins, are enriched in the target genes’ promoter, facilitating WRKYs’ interaction and subsequent regulation of gene expression [24,26]. Promoter analysis showed that this enrichment is a common feature among the promoters of various Al resistance genes, such as *MATE*, *STAR1*, *ALS1* and so on [20,27]. Evidence from Ding’s study suggests that WRKY46, another distinct WRKY transcription factor, directly associates with the *ALMT1* promoter by recognizing specific W-box sequences. Consistent with elevated *ALMT1* expression, the *wrky46-1* mutant shows increased malate secretion from its roots and significantly stronger resistance to aluminum toxicity. Induction of the *WRKY46* has been observed under diverse conditions, including exposure to abiotic stresses like salt, drought, and UV-B, and biotic stresses such as salicylic acid or infection with biotrophic pathogens [20]. Our results clearly demonstrate that WRKY61 also binds to the W-BOX motif within the *ALMT1* promoter region and suppresses its expression, thereby participating in the plant’s response to aluminum stress. Different WRKY members respond to distinct environmental signals yet converge on the same biological process. Both WRKY61 and WRKY46 directly bind the ALMT1 promoter and repress its expression, potentially coordinating the trade-off between plant growth and stress defense in response to divergent environmental cues.

## 4. Materials and Methods

### 4.1. Plant Materials and Growth Conditions

*Arabidopsis thaliana* Columbia-0 (Col), *wrky61-1* mutant (SALK_006029C), and *wrky61-2* mutant (GABI_561F07), obtained from the NASC stock center, were used in this study. Surface-sterilized seeds were sown on 1/2 Murashige and Skoog (MS) medium containing 1% sucrose and 0.6% agar. Plates were then transferred to a growth chamber set to 22 ± 1 °C, with a 16 h light/8 h dark photoperiod and a light intensity of 80–90 μmol m^−2^s^−1^, to allow for germination and seedling growth.

### 4.2. Histochemical Staining and Cytological Observation

For histochemical staining, 5-day-old Arabidopsis seedlings with 1 cm-long roots were incubated in either DAB (0.1% DAB in 50 mM Tris-HCl, pH 5.0) or NBT (0.1% NBT in phosphate buffer, pH 7.0) staining solution for 30 min. The staining duration was adjusted as needed to achieve optimal color development. For Trypan blue staining, roots were immersed in a 0.4% solution for 5 min. Hydrogen peroxide (H_2_O_2_) levels were detected by DAB staining, superoxide anion (O_2_^−^) content by NBT staining, and cell viability was assessed using Trypan Blue staining. Following all staining procedures, seedlings were mounted on glass slides in HCG solution (24 g chloral hydrate, 3 mL glycerol, 9 mL H_2_O). Samples were then observed and imaged using a Leica DM2500 microscope. Staining intensity was quantified with Image J (v1.54r). Given the variability associated with reagents and staining protocols, it is important to note that DAB and NBT staining provide only a relative measure of ROS levels, suitable for comparison within this study.

### 4.3. Gene Expression Analysis

Total RNA was extracted from 10-day-old Arabidopsis seedlings or roots of Arabidopsis using the TaKaRa MiniBEST Plant RNA Extraction Kit, following the manufacturer’s protocol (TaKaRa, Dalian, China). Subsequently, cDNA was synthesized from the total RNA using the PrimeScript™ 1st Strand cDNA Synthesis Kit (TaKaRa). Real-time quantitative PCR (qPCR) was performed using a Thermo Fisher QuantStudio 3 system and the SYBR Premix Ex Taq™ II kit (TaKaRa), following the manufacturer’s protocol. Transcript levels were calculated using the ΔCt method and normalized to the *ACTIN2* reference gene. Gene-specific primers are listed in Table S1.

### 4.4. Quantification of Malate Secretion by HPLC-MS

Organic acid anions, retained on a Dowex 1 × 8 resin, were eluted with 15 mL of 1 M HCl. The resulting eluate was then concentrated to dryness under reduced pressure at 40 °C using a rotary evaporator. Chromatographic separation was performed on an XBridge C18 column (4.6 mm × 250 mm, 5 µm), which was maintained at 40 °C. The mobile phase consisted of acetonitrile (A) and 0.1% aqueous phosphoric acid (B), delivered at a flow rate of 1.0 mL/min. A gradient elution program was employed as follows: 5% A (0–15 min), linearly increased to 80% A (15–22 min), held at 80% A (22–30 min), and then returned to 5% A for column re-equilibration (30–40 min). The injection volume was 5 µL, and detection was carried out at a wavelength of 285 nm.

### 4.5. Dual-Luciferase Assay

To construct the effector vector, the full-length coding sequence (CDS) of *WRKY61* was amplified and cloned into the binary vector pCambia1300, downstream of the *35S* promoter. The intact 1500 bp *ALMT1* promoter was amplified by PCR, while a W-box-deleted variant was synthesized, and both constructs were subsequently cloned into the reporter vector pGreenII0800-LUC[33]. The recombinant plasmids were transformed into Agrobacterium EHA105 by co-transfer with the pSoup plasmid, which is necessary for plasmid maintenance. Co-infiltration of the EHA105 lines into *N. benthamiana* leaves was carried out as described by Yang et al. [34]. Luciferase activities (Firefly and Renilla) were then measured using a Dual Luciferase Assay Kit (Promega (Madison, WI, USA)), with all primer sequences listed in Table S1.

### 4.6. Yeast One-Hybrid Assay

The WRKY61 coding region was amplified and cloned into the pGADT7-rec2 prey vector, generating a translational fusion with the GAL4 activation domain. For pHIS2 vector construction, the intact P1 and W-box-deleted P2 ALMT1 promoter fragments were cloned and ligated into the vector. For yeast transformation, 50 μL of Y187 competent cells were incubated with 100 ng each of pHIS2 bait and pGADT7-Rec2 prey vectors, 50 μg salmon sperm carrier DNA, and 0.5 mL PEG/LiAc solution. Co-transformants were selected on SD/–Leu/–Trp plates at 28 °C for 4 days. Selected colonies were grown in SD/–Leu/–Trp liquid medium to OD_600_ = 0.1, then 5 μL was spotted onto SD/–Leu/–Trp control plates and SD/–His/–Leu/–Trp plates supplemented with 50 mM 3-amino-1,2,4-triazole (3-AT). Plates were incubated at 28 °C for 3 days.

### 4.7. Determination of Al Content in Plants

Ten-day-old Arabidopsis thaliana Col–0 and wrky61–1 seedlings were transferred to either fresh nutrient solution (control) or 0.5 mM CaCl_2_ solution. Subsequently, plants were exposed to 200 μM AlCl_3_ for 24 h or 48 h. Seedlings were harvested, weighed, and digested in HNO_3_:HClO_4_ (4:1, *v*/*v*). Aluminum content in the digests was quantified via inductively coupled plasma-atomic emission spectrometry (ICP-AES).

## 5. Conclusions

In this research study, a screen for Arabidopsis mutants with altered aluminum resistance identified a loss-of-function mutant of the WRKY61 transcription factor that exhibits significantly enhanced resistance to aluminum toxicity. This finding indicates that WRKY61 is a negative regulator of the plant’s response to aluminum toxicity. Further analysis revealed that WRKY61 directly represses *ALMT1* expression by binding to W-box elements in its promoter. The *wrky61-1* mutant’s heightened resistance to aluminum toxicity is a direct consequence of derepressed *ALMT1* expression. In the absence of functional WRKY61, *ALMT1* transcription is no longer inhibited, leading to increased malate secretion from the roots. This demonstrates that WRKY61 is a negative regulator of aluminum resistance, functioning as a transcriptional repressor of *ALMT1*.

## Data Availability

The original contributions presented in this study are included in the article. Further inquiries can be directed to the corresponding authors.

## Supplementary Materials

The following supporting information can be downloaded at: https://www.mdpi.com/article/10.3390/plants14213286/s1, Figure S1. wrky61-2 mutant exhibited enhanced aluminum resistance. (A) Schematic diagram of *wrky61-2* mutants. (B) Semi-quantitative PCR was performed to detect the expression of WRKY61 in the *wrky61-2* mutant, with *ACTIN2* as the internal reference gene. (C) Phenotypes of wild-type (Col) and *wrky61-2* mutant plants grown on solid medium with or without 200 µM AlCl_3_ treatment (pH 5.0). Scale bar = 5 mm. CK, the blank control. (D) The length of root of the seedling in (C). Data are presented as the mean ± SE (n > 20). Asterisks indicate statistically significant differences (***, *p* < 0.01). Table S1. Primers used in the study.

## Abbreviations

The following abbreviations are used in this manuscript:

WRKY61	WRKY DNA-BINDING PROTEIN 61
ALMT1	ALUMINUM-ACTIVATED MALATE TRANSPORTER 1
STOP1	SENSITIVE TO PROTON RHIZOTOXICITY 1
MATE	MULTI-DRUG AND TOXIC COMPOUND EXTRUSION
RAE1	REGULATION OF ATALMT1 EXPRESSION 1
CAMTA2	CALMODULIN-BINDING TRANSCRIPTION ACTIVATOR 2
CML24	CALMODULIN-LIKE 24
