# Supplementary material for: WRKY61 Negatively Regulates Aluminum Resistance by Inhibiting the Expression of ALMT1 in Arabidopsis thaliana
Source: Plants (Basel). 2025 Oct 27;14(21):3286. doi: 10.3390/plants14213286 (PMC12610487; doi:10.3390/plants14213286)
Supplement: Supplementary file 1 [file plants-14-03286-s001.zip › plants-3823667-supplementary.pdf]

Figure S1

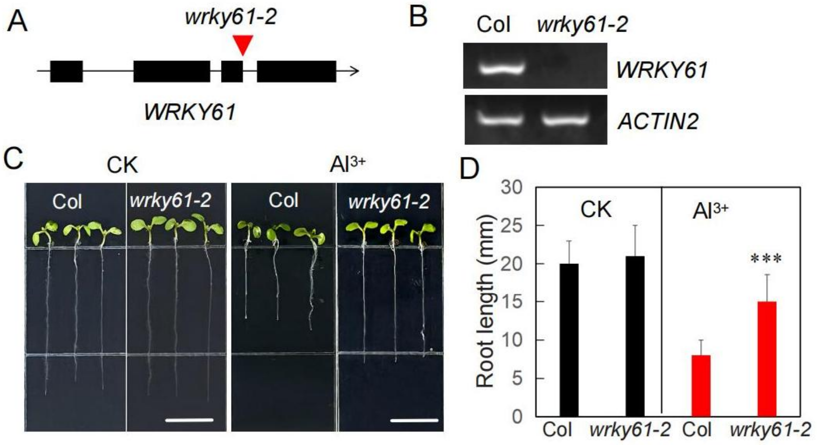

Figure S1. *wrky61-2* mutant exhibited enhanced aluminum resistance. (A) Schematic diagram of *wrky61-2* mutants. (B) Semi-quantitative PCR was performed to detect the expression of WRKY61 in the *wrky61-2* mutant, with *ACTIN2* as the internal reference gene. (C) Phenotypes of wild-type (Col) and *wrky61-2* mutant plants grown on solid medium with or without 200 μM $AlCl_3$ treatment (pH 5.0). Scale bar = 5 mm. CK, the blank control. (D) The length of root of the seedling in (C). Data are presented as the mean ± SE (n >20). Asterisks indicate statistically significant differences (***, P <0.01).

Table S1. Primers used in the study.

Name	Sequences	Process
wrky61-1-F	GTTGAAGAAGCGGAAGGT	Mutant identification
wrky61-1-R	GATCTCACCGAAACCCTA	Mutant identification
WRKY61-RT-F	CTCATCAAGAACACGACCAT	Semi-quantitative PCR
WRKY61-RT-R	TCTGTCACCTTCCGCTTC	Semi-quantitative PCR
ACTIN2-RT-F	GCTCCTCTTAACCCAAAGGC	Semi-quantitative PCR
ACTIN2-RT-R	CACACCATCACCAGAATCCAGC	Semi-quantitative PCR
WRKY61-Q-F	AAACTAGGGTTTCGGTGAG	Real-time qPCR
WRKY61-Q-R	ACAAGAAGCTGCAATGGT	Real-time qPCR
ALMT1-Q-F	ACTTGAGAGAGCTGAGTGACC	Real-time qPCR
ALMT1-Q-R	TCTTCTCGGGTCTTCATTCCC	Real-time qPCR
MATE-Q-F	GCATAGGACTTCCGTTTGTGGCA	Real-time qPCR
MATE-Q-R	CGAACACAAACGCTAAGGCA	Real-time qPCR
STOP1-Q-F	CCAAGTTCCATCTCAAGCTTTTCT	Real-time qPCR
STOP1-Q-R	TGGGACGTAAAACCTGCGAA	Real-time qPCR
ACT2-Q-F	GCTGACCGTATGAGCAAAGA	Real-time qPCR
ACT2-Q-R	GATCCACATCTGTTGGAACG	Real-time qPCR
WRKY61-PROMOTER-F	CATGTATATCATAGCTGGAGTCA	vector construction
WRKY61-PROMOTER-R	ATTAGTCTTCTTTGATAAATTTTTT	vector construction
WRKY61-CDS-F	ATGGAGAAGGACGATTTCTTG	vector construction
WRKY61-CDS-R	TTAAGGGCTCTTCTCAGCTTC	vector construction
